# Regulatory T cells induce a suppressive immune milieu and promote lymph node metastasis in intrahepatic cholangiocarcinoma

**DOI:** 10.1038/s41416-022-01838-y

**Published:** 2022-05-21

**Authors:** Daisuke Konishi, Yuzo Umeda, Kazuhiro Yoshida, Kunitoshi Shigeyasu, Shuya Yano, Tomohiro Toji, Sho Takeda, Ryuichi Yoshida, Kazuya Yasui, Tomokazu Fuji, Kazuyuki Matsumoto, Hiroyuki Kishimoto, Hiroyuki Michiue, Fuminori Teraishi, Hironari Kato, Hiroshi Tazawa, Hiroyuki Yanai, Takahito Yagi, Ajay Goel, Toshiyoshi Fujiwara

**Affiliations:** 1 Department of Gastroenterological Surgery and Surgical Oncology, Okayama University Graduate School of Medicine, Dentistry and Pharmaceutical Sciences, Okayama, Japan; 2 Department of Diagnostic Pathology, Okayama University Hospital, Okayama, Japan; 3 Department of Gastroenterology and Hepatology, Okayama University Graduate School of Medicine, Dentistry and Pharmaceutical Sciences, Okayama, Japan; 4 Neutron Medical Research Center, Okayama University Graduate School of Medicine, Dentistry and Pharmaceutical Sciences, Okayama, Japan; 5 Department of Molecular Diagnostics and Experimental Therapeutics, Beckman Research Institute of City of Hope, Duarte, CA USA

**Keywords:** Liver cancer, Tumour immunology

## Abstract

**Background:**

Emerging evidence indicates that immunogenicity plays an important role in intrahepatic cholangiocarcinoma (ICC). Herein, we systematically evaluated the clinical relevance of immunogenicity in ICC.

**Methods:**

Highly immunogenic ICCs identified in the public dataset and the Cancer Immunome Atlas (TCIA) were assessed to determine the prognostic impact of immunogenicity in ICC and key components after curative resection. We also investigated the clinical relevance of the immune milieu in ICC.

**Results:**

Using the Gene Expression Omnibus dataset 89749 and TCIA, we identified CD8^+^/forkhead box P3 (FoxP3)^+^ tumour-infiltrating lymphocytes (TILs), T-cell immunoglobulin and mucin domain 3 (TIM-3) and human leukocyte antigen-A (HLA-A) in highly immunogenic ICCs. Immunohistochemical analysis of the in-house cohort showed that intratumoral FoxP3^+^ TILs correlated with CD8^+^ TILs (*P* = 0.045, Fisher’s exact test) and that high FoxP3^+^/CD8^+^ ratio (FCR) was an important marker for poor survival (*P* < 0.001, log-rank test). Furthermore, the FCR was higher in tumour-free lymph nodes in ICCs with lymph node metastases than in those without lymph node metastases (*P* = 0.003, Mann–Whitney *U* test).

**Conclusions:**

FCR should be considered an important biomarker that represents the immune environment of ICC based on its potentially important role in tumour progression, especially lymph node metastasis.

## Background

The incidence of intrahepatic cholangiocarcinoma (ICC), which accounts for 10–20% of all primary liver cancers, is increasing worldwide [1, 2]. Surgical tumour resection is the only treatment for a potential cure; however, the prognosis of ICC after surgery remains dismal, with 5-year overall survival (OS) rates of 30–40%, which is primarily due to high recurrence rates and lack of systemic therapeutic options after recurrence [3]. Thus, the development of novel treatment strategies and clarification of the underlying tumour biology are urgently needed to improve patient survival.

Uncovering the role of tumour immunogenicity in many malignancies has led to the development of immunotherapeutic strategies, which are considered a novel treatment option in ICC as well [4]. Tumour immunogenicity is defined as the ability of a tumour to induce an immune response that can prevent its growth [5]. A tumour harbouring a large number of somatic mutations, i.e. with a high tumour mutation burden (TMB), tends to induce an immune response and is considered a highly immunogenic [4]. Melanoma, lung cancer and mismatch repair-deficient colorectal cancer possess a high TMB, whereas pancreatic cancer, myeloma, and mismatch repair-proficient colorectal cancer harbour a low TMB [4, 6]. However, even in mismatch repair-proficient colorectal cancer, high densities of CD3^+^, CD8^+^ and CD45RO^+^ tumour-infiltrating lymphocytes (TILs) are observed in around 50% of the tumours [7], indicating that even some tumours harbouring a low TMB can induce an immune response. Similarly, although ICCs harbour a relatively low TMB (median, 1.9/Mb; range, 0.5–11.0/Mb) [8], some studies have reported increased density of CD8^+^ TILs in ICCs [9–12], and RNA sequencing analysis have revealed that one of the ICC subtypes can be characterised by immune-related pathways [13]. However, the characteristics of highly immunogenic ICCs and the mechanism by which the immune milieu regulates ICC biology remain unclear.

Several bioinformatics approaches, including single-cell and bulk RNA sequencing, have enabled the identification of immune cell populations within the immune milieu [14, 15]. Aaron et al. have developed the CIBERSORT method to dissect the tumour immune milieu, enabling the characterisation of immune cell fractions based on gene expression analysis of bulk RNA sequencing data [14]. This method has overcome the disadvantages of single-cell RNA sequencing, which is challenging for its application in clinical use. Furthermore, Charoentong et al. utilised advances in bioinformatics and machine learning approaches to develop an immunophenogram and The Cancer Immunome Atlas (TCIA) (https://tcia.at/) based on data from The Cancer Genome Atlas and completed the immunogenic characterisation of 20 solid tumours [16].

To elucidate the characteristics of highly immunogenic ICCs and the relationship of tumour immunity with tumour biology, we performed a comprehensive analysis of the immune milieu in ICC. First, we characterised highly immunogenic ICCs based on metagene analysis using a publicly available dataset and TCIA. Second, we assessed the prognostic impact of tumour immunity after liver resection in patients with ICCs. Finally, we determined the clinical relevance of the immune milieu in ICC.

## Materials and methods

### Samples and study design

A total of 61 consecutive patients who underwent curative hepatic resection for ICC between January 1, 1999 and December 31, 2015 in Okayama University Hospital were enrolled in this study. The study samples comprised primary lesions from 61 patients with ICC and corresponding lymph nodes from 47 of the 61 patients. The baseline clinicopathological variables, including age, sex, aetiology, location, macroscopic morphology, differentiation, tumour size, serosal invasion, tumour number, preoperative carbohydrate antigen 19-9 and carcinoembryonic antigen levels and tumour stage are summarised in Table 1. The Tumor-Node-Metastasis staging system from 7th edition of the American Joint Committee on Cancer was used for the pathological staging of the cases [17]. During annual hospital visits, all patients underwent extensive evaluation including blood tests, chest X-ray, abdominal computed tomography or magnetic resonance imaging studies. The need for informed consent was waived due to the retrospective study design. The institutional review boards of all participating institutions approved the study (no. 1911-032).

**Table 1:** Patient characteristics.

Variables	
Age median, (range)	67 (45–85)
Sex	Female (*N* = 27)
	Male (*N* = 34)
HBs-Ag	Positive (*N* = 7)
	Negative (*N* = 53)
HCV-Ab	Positive (*N* = 5)
	Negative (*N* = 55)
Morphology	MF (*N* = 39)
	MF + PI (*N* = 22)
Differentiation	Well (*N* = 9)
	Moderate (*N* = 39)
	Poor (*N* = 9)
	Others (*N* = 2)
Tumour location	Hilar (*N* = 36)
	Peripheral (*N* = 25)
Tumour size	≦5 cm (*N* = 34)
	>5 cm (*N* = 27)
Tumour number	Single (*N* = 44)
	Multiple (*N* = 17)
Vascular invasion^a^	Positive (*N* = 32)
	Negative (*N* = 16)
Serosal invasion	Positive (*N* = 30)
	Negative (*N* = 31)
Lymph node metastasis	Presense (*N* = 23)
	Absense (*N* = 25)
	Undetermined (*N* = 13)
Distant metastasis	Presence (*N* = 4)
	Absence (*N* = 57)
Preoperative	≦37 U/ml (*N* = 23)
Serum CA19-9	>37 U/ml (*N* = 29)
Preoperative	≦5 ng/ml (*N* = 38)
Serum CEA	>5 ng/ml (*N* = 15)
Hepatectomy	(Extended) Segmentectomy (*N* = 5)
	(Extended) hepatic lotectomy (*N* = 48)
	Trisegmentectomy (*N* = 8)
Lymphadenectomy	Performed (*N* = 47)
	Unperformed (*N* = 14)
Curability	R0 (*N* = 58)
	R1 (*N* = 3)
Observation time, (days), median, (range)	824, (38–5486)

*CA19-9* carbohydrate antigen 19-9, *CEA* carcinoembryonic antigen, *MF* mass forming type, *PI* periductal infiltrating type.

^a^13 patients records were not available.

The study comprised an initial discovery phase followed by an independent clinical validation phase. In the discovery phase, we identified highly immunogenic ICCs and deregulated immune components using the publicly available Gene Expression Omnibus (GEO) microarray database (GSE 89749) [13] and the immunophenogram [16]. In the clinical validation phase, we evaluated the clinical relevance of highly immunogenic ICCs in the in-house cohort using immunohistochemical analysis.

### Sequential double-staining immunohistochemistry and single staining immunohistochemistry

We performed double-staining immunohistochemistry for evaluation of the phenotypes of effector and suppressor cells in the immune milieu. In brief, 4 µm-thick sections of representative blocks were deparaffinised and dehydrated using gradient solvent washes. Following endogenous peroxidase blockade using 3% H_2_O_2_ for ten minutes, antigen retrieval was performed with ethylene-diamine-tetraacetic acid buffer (pH 9.0). Thereafter, the slides were incubated with Protein Block Serum-Free Ready-to-Use (Agilent Technologies, Santa Clara, CA, USA) for ten minutes followed by anti-CD8a antibody or anti-FoxP3 antibody (Supplementary Table 1) at room temperature for one hour. After rinsing with PBS for 5 min three times, the slides were incubated with EnVision+ System-HRP Labelled Polymer Anti–mouse (Agilent Technologies, Santa Clara, CA, USA) for 30 min, followed by rinse with phosphate-buffered saline (PBS) for five minutes three times. Liquid DAB + Substrate Chromogen System (Agilent Technologies, Santa Clara, CA, USA) was used as a chromogen. Then, following antigen retrieval in ethylene-diamine-tetraacetic acid buffer (pH 9.0), the slides were incubated with Protein Block Serum-Free Ready-to-Use for 10 min and anti-PD1 antibody and anti-CD4 antibody (Supplementary Table 1) overnight for slides incubated with anti-CD8a antibody and anti-FoxP3 antibody, respectively. Next, the slides were incubated with EnVision+ System-HRP Labelled Polymer Anti–Rabbit (Agilent Technologies, Santa Clara, CA, USA), followed by incubation with Histogreen, Substrate kit for peroxidase (Takara Bio Inc., Shiga, Japan) and hematoxylin as a nuclear counterstain.

In addition, to evaluate the immune milieu in ICC, we determined the numbers and distribution of effector and suppressor cells as well as the intensity of immune checkpoint molecules and human lymphocyte antigens (HLAs) using immunohistochemistry. Manual immunostaining was performed using formalin-fixed paraffin-embedded tissues. Briefly, 4 µm-thick sections of representative blocks were deparaffinised and dehydrated using gradient solvent washes. Following endogenous peroxidase blockade using 3% H_2_O_2_ for 10 min, antigen retrieval was performed with ethylene-diamine-tetraacetic acid buffer (pH 9.0). Thereafter, the slides were incubated with indicated primary antibodies (Supplementary Table 1) at room temperature for one hour. Next, the slides were incubated with appropriate secondary antibodies and the avidin–biotin–peroxidase complex (Vector Laboratories, Burlingame, CA, USA), followed by incubation with biotinyl tyramide and streptavidin peroxidase (Agilent Technologies, Santa Clara, CA, USA). Diaminobenzidine (Takara Bio Inc., Shiga, Japan) was used as a chromogen and hematoxylin as a nuclear counterstain.

The slides were evaluated under the guidance of a pathologist without reference to the patient’s clinical profile. First, we defined intratumoral and stromal TILs as lymphocytes within the tumour nest or in direct contact with tumour cells and within the tumour stroma, respectively, following the recommendations of the International TILs Working Group 2014 [18]. Second, for all ICCs, intratumoral and stromal TIL counts within the borders of the tumour were calculated in four independent fields visualised at ×400 magnification. Finally, averaged intratumoral or stromal TIL counts in four independent fields used as representative TILs in a tumour for further analysis. Cut-off values for effector and suppressor cell counts were determined using the X-tile analysis (New Haven, CT, USA) of overall survival (OS) [19]. The expression status of immune checkpoint molecules and major histocompatibility complex (MHC)-related proteins were evaluated using the immunoreactive score, which is based on the percentage of positive lymphocytes or tumour cells and the intensity of expression [20]. As described previously, the percentage of positive cell staining was scored as follows: 1, 0–10%; 2, 11–50%; 3, 51–80% and 4, 81–100%. Staining intensity was scored as follows: 1, weak; 2, moderate and 3, intense. The immunoreactive score was used to categorise the strength of protein expression into four subsets as follows: 0–1, no staining; 2–3, weak staining; 4–8, moderate staining and 9–12, strong staining.

### Statistical analysis

All statistical analyses were performed using EZR (Saitama Medical Center, Jichi Medical University) [21], which is a graphical user interface for R (The R Foundation for Statistical Computing, version 2.13.0), and plotted using GraphPad Prism version 9 (GraphPad Software, San Diego, CA, USA). Categorical variables were compared using Fisher’s exact test. Differences between continuous variables were determined using the Mann–Whitney *U* or Wilcoxon signed-rank test. The correlation was estimated using Spearman’s rank correlation coefficient. OS was calculated from the date of surgical resection to the date of death due to ICC or last follow-up for censored patients. OS was estimated using the Kaplan–Meier method and compared by the log-rank test. All *P*-values were calculated using two-sided tests, and a *P-*value of < 0.05 was considered to indicate statistical significance.

## Results

### Identification of immune components associated with highly immunogenic ICC

**Fig. 1:**
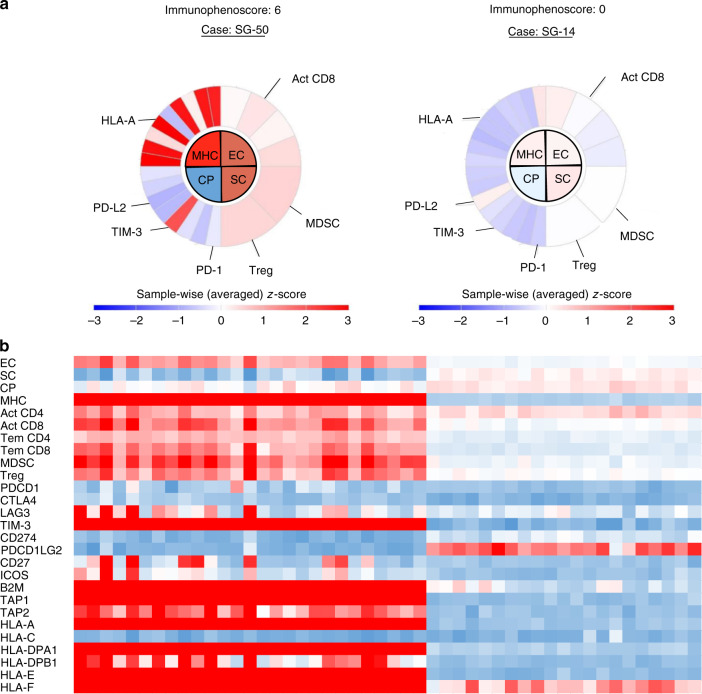
Immunophenogram analysis reveals the characteristics of highly immunogenic ICCs. **a** Representative immunophenograms of highly or weakly immunogenic ICCs based on metagene analysis using the TCIA. Immunophenoscore ranges from 0 to 10 scale based on the sum of the weighted average z-score of the four categories. Act CD4/CD8 activated CD4 ^+^ /CD8 ^+ ^T cells, B2M beta-2 microglobulin, CP checkpoints/immunomodulators, CTLA4 cytotoxic T-lymphocyte-associated protein 4, EC effector cells, HLA human leukocyte antigen, IOCS induced T-cell co-stimulator, LAG3 lymphocyte activation gene 3, MDSC myeloid-derived suppressor cells, MHC major histocompatibility complex, PDCD1 programmed cell death 1, PDCD1LG2 programmed cell death 1 ligand 2, SC suppressor cells, TAP1/2 transporter associated with antigen processing 1/2, Tem CD4/CD8 cells effector memory CD4^+^/CD8^+^ T cells, TIM-3 T-cell immunoglobulin and mucin domain-containing protein 3, Treg regulatory T cells. **b** Heatmap of 48 fluke-negative ICCs. Highly immunogenic ICCs were characterised based on effector cells, suppressor cells and MHC-related molecules. ICC intrahepatic cholangiocarcinoma.

To characterise the immune milieu in ICC, we first performed a comprehensive analysis of the ICC immunogenicity using the RNA sequencing data from the publicly available GSE 89749 dataset [13], which comprised the RNA sequencing results of bulk tissue samples from 92 fluke-positive and 48 fluke-negative ICCs. Fluke-negative ICCs are more frequent in developed countries, and all cases examined in subsequent analyses in the current cohort were fluke-negative ICCs [22]. Therefore, we assessed the immune milieu of 48 fluke-negative ICCs using the TCIA calculator (https://tcia.at/) and determined the immunophenoscore of each ICC as an indicator of immunogenicity based on the z-score of four immune components, namely effector cells, suppressor cells, immune checkpoint molecules, and MHC-related proteins. Figure 1a shows representative immunophenoscores of ICCs with high and low immunogenicity. According to the immunophenoscore, we classified the ICCs into high and low immunogenicity groups (Fig. 1b). Highly immunogenic ICCs were characterised by high numbers of effector and suppressor cells within the entire tumour. Additionally, the highly immunogenic ICCs expressed high levels of MHC-related molecules compared to the weakly immunogenic ICCs and expressed low levels of immune checkpoint molecules except for T-cell immunoglobulin and mucin domain 3 (TIM-3). These findings suggested that suppressor cells induced immune tolerance in highly immunogenic ICCs by suppressing the activity of effector cells. To further analyse the suppressive immune milieu in highly immunogenic ICCs, we focused on activated CD8^+^ tumour-infiltrating lymphocytes (TILs) as effector cells, tumour-infiltrating regulatory T cells (Treg) as suppressor cells, TIM-3 as an immune checkpoint molecule and HLA-A, based on the immunophenoscore analysis.

### CD8^+^/FoxP3^+^ TILs and TIM-3 and HLA- A protein expression in the local immune milieu of highly immunogenic ICCs

Not only the immunogenicity of the entire tumour but also the local immune response to the tumour can impact tumour biology such as that observed in breast cancer [23]. To elucidate the local immune milieu in ICC, we first evaluated the phenotypes of stromal and intratumoral TILs in ICCs. Using enzymatic double-staining immunohistochemistry, we analysed CD8 ^+^ PD-1^+^ TILs and CD4 ^+^ FoxP3^+^ TIL counts in the stroma and intra-tumour in nine ICC patients selected based on high TILs in hematoxylin and eosin staining (Supplementary Fig 1). Median stromal and intratumoral CD8^ +^ PD-1^+^ TILs in ICC accounted for 1.1% (IQR 0.4–3.3%) and 2.0% (IQR 0.3–3.6%) of CD8^+^ TILs, respectively. Because CD8^+^ PD-1^+^ TILs, exhausted CD8 TILs, were much less observed compared to CD8^+^ PD-1^−^ TILs, CD8 could be used as a marker of activated CD8^+^ TILs in ICC. In addition, stromal and intratumoral FoxP3^+^ CD4^+^ TILs in ICC accounted for almost 100% of FoxP3^+^ TILs, indicating that FoxP3 could be used as a marker of Treg TILs. Based on these results, for further analysis, we used CD8 and FoxP3 as a marker of activated CD8^+^ and Treg TILs in ICCs, respectively.

**Fig. 2:**
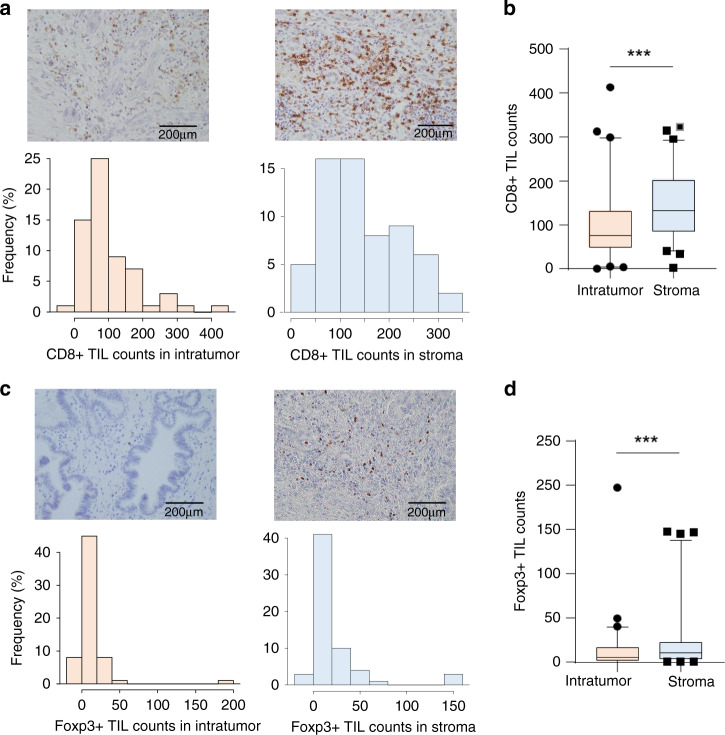
Immunohistochemical analysis reveals the characteristics of local immune milieu of highly immunogenic ICCs. **a** Upper images show representative CD8^+^ TIL counts in ICCs. Left side shows low counts of CD8^+^ TILs and right side high counts of infiltrating TILs. Lower bar graphs show the distribution of CD8^+^ TIL counts in intra-tumour and stroma of ICC. **b** Boxplot showing that CD8^+^ TIL counts are higher in the stroma than in the tumour. The upper and lower bars represent 5% and 95%, respectively. ***P < 0.001. **c** Upper images show representative FoxP3^+^ TIL counts in ICCs. Left side shows low counts of FoxP3^+^ TILs and right side high counts of infiltrating TILs. Lower bar graphs show the distribution of FoxP3^+^ TIL counts in intra-tumour and stroma of ICC. **d** Boxplot showing that FoxP3^+^ TIL counts are higher in the stroma than in the tumour. The upper and lower bars represent 5% and 95%, respectively. ***P < 0.001.

Then, we determined the number of stromal and intratumoral CD8^+^ and FoxP3^+^ TILs in the same flied using immunohistochemistry in the clinical validation cohort. The median number of CD8^+^ TILs was significantly higher in the stroma than in the intratumoral area in the same section (132/slide [range, 1.25–321.75] versus 76.25/slide [range, 0–411.75], *P* < 0.001, Wilcoxon signed-rank test; Fig. 2a and b). Similarly, the median number of infiltrating FoxP3^+^ TILs was higher in the stroma than in the intratumoral area in the same section (10.7/slide [range, 0–147.5] versus 5.5/slide [range, 0–197.5], *P* < 0.001, Wilcoxon signed-rank test; Fig. 2c and d).

**Fig. 3:**
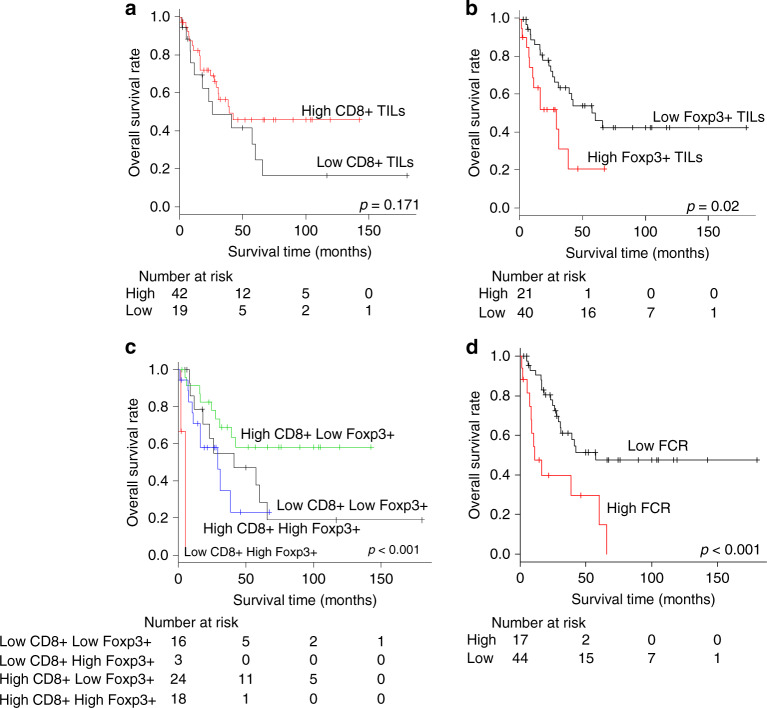
Prognostic analysis with tumour-infiltrating lymphocytes (TILs) in intra-tumour of ICCs. **a** CD8^+^ TILs, **b** FoxP3^+^ TILs, **c** combination of CD8^+^ and FoxP3^+^ TILs and **d** FoxP3^+^/CD8^+^ TIL ratio.

Next, we determined the optimal cut-off for CD8^+^/FoxP3^+^ TIL counts in intratumoral and stromal areas of highly immunogenic ICCs using the X-tile analysis of OS: intratumoral CD8^+^ TILs, 53.8; stromal CD8^+^ TILs, 80.25; intratumoral Foxp3^+^ TILs, 11.75; stromal Foxp3^+^ TILs, 5.75. We found no significant differences in OS between ICCs with high and low CD8^+^ TIL counts in the intratumoral and stromal areas (*P* = 0.171 and 0.133, respectively, log-rank test; Fig. 3a and Supplementary Fig. 2A). The OS rate of ICCs with high intratumoral FoxP3^+^ TIL counts was significantly worse than that of ICCs with low intratumoral FoxP3^+^ TIL counts (*P* = 0.02, log-rank test, Fig. 3b); however, there was no significant difference in the OS between ICCs with high and low intrastromal FoxP3^+^ TIL counts (*P* = 0.254, log-rank test, Supplementary Fig. 2B). While there was no correlation between the number of CD8^+^ TILs and that of FoxP3^+^ TILs in the stroma (*P* = 1, Fisher’s exact test), CD8^+^ TIL counts were significantly correlated with FoxP3^+^ TIL counts in the intratumoral area (*P* = 0.046, Fisher’s exact test, Supplementary Table 2). These results indicated that the immune milieu might be suppressed by the accumulation of FoxP3^+^ TILs in the intra-tumour but not in the stroma of highly immunogenic ICCs.

Finally, we assessed the intratumoral expression levels of TIM-3 and HLA-A in ICC using immunohistochemistry. While moderate or intense HLA-A expression was observed in ~66% of the ICCs, 54% of the examined ICCs exhibited absent or weak TIM-3 expression (Supplementary Fig. 3 and Supplementary Table 2). There was no correlation between CD8^+^ TIL counts and TIM-3 expression (*P* = 0.356, Fisher’s exact test); however, there was a trend of correlation between CD8^+^ TIL counts and HLA-A expression (*P* = 0.075, Fisher’s exact test). These results indicated that FoxP3^+^ TILs might mainly function as suppressors within the tumour, which is a characteristic of highly immunogenic ICCs.

### Role of the FoxP3^+^/CD8^+^ ratio in the OS of ICC

We elucidated the prognostic impact of ICC immune milieu by comparing OS among ICCs classified into four groups based on the number of intratumoral CD8^+^ and FoxP3^+^ TILs. As expected, the patients with ICC harbouring high CD8^+^ TIL and low FoxP3^+^ TIL counts in the intra-tumour exhibited the most favourable OS whereas those with low CD8^+^ TIL and high FoxP3^+^ TIL counts in the intra-tumour exhibited the worst OS (*P* < 0.001, log-rank test; Fig. 3c). In addition, we investigated whether the balance between intratumoral effector and suppressor cells had an impact on the OS of patients with ICC. To that end, we used the FoxP3^+^/CD8^+^ TIL ratio (FCR) to classify patients into low and high-FCR groups using the cut-off value generated using X-tile analysis: the cut-off value of FCR was 0.22. Interestingly, the OS of high-FCR ICCs was worse than that of low-FCR ICCs (*P* < 0.001, log-rank test; Fig. 3d). While high-FCR ICCs significantly associated with pathological lymph node metastasis (*P* = 0.045, Fisher’s exact test; Table 2), they did not have a correlation with other clinicopathological factors, such as tumour location and the tumour makers.

**Table 2:** Correlation between clinicopathological variables and FCR.

Variables		FCR low	FCR high	*P*-value
		(*N* = 44, 72.1%)	(*N* = 17, 27.9%)	
Age median, (range)	67 (45–85)	67.5 (45–85)	67.0 (51–83)	0.750^b^
Sex	Female (*N* = 27)	19 (70.4)	8 (29.6)	1
	Male (*N* = 34)	25 (73.5)	9 (26.5)	
HBs-Ag	Positive (*N* = 7)	4 (57.1)	3 (42.9)	0.393
	Negative (*N* = 53)	39 (73.6)	14 (26.4)	
HCV-Ab	Positive (*N* = 5)	4 (80.0)	1 (20.0)	1
	Negative (*N* = 55)	39 (70.9)	16 (29.1)	
Morphology	MF (*N* = 39)	29 (74.4)	10 (25.6)	0.767
	MF + PI (*N* = 22)	15 (68.2)	7 (31.8)	
Differentiation	Well (*N* = 9)	8 (88.9)	1 (11.1)	0.423
	Moderate (*N* = 39)	25 (64.1)	14 (35.9)	
	Poor (*N* = 9)	7 (77.8)	2 (22.2)	
	Others (*N* = 2)	2 (100.0)	0 (0.0)	
Tumour location	Hilar (*N* = 36)	27 (75.0)	9 (25.0)	0.574
	Peripheral (*N* = 25)	17 (68.0)	8 (32.0)	
Tumour size	≦5 cm (*N* = 34)	26 (76.5)	8 (23.5)	0.566
	>5 cm (*N* = 27)	18 (66.7)	9 (33.3)	
Tumour number	Single (*N* = 44)	32 (72.7)	12 (27.3)	1
	Multiple (*N* = 17)	12 (70.6)	5 (29.4)	
Vascular invasion^a^	Positive (*N* = 32)	22 (68.7)	10 (31.3)	0.746
	Negative (*N* = 16)	12 (66.7)	4 (33.3)	
Serosal invasion	Positive (*N* = 30)	20 (77.4)	7 (22.6)	0.402
	Negative (*N* = 31)	24 (66.7)	10 (33.3)	
Lymph node metastasis	Presense (*N* = 23)	14 (60.9)	9 (39.1)	0.045
	Absense (*N* = 25)	22 (88.0)	3 (12.0)	
	Undetermined (*N* = 13)			
Distant metastasis	Presense (*N* = 4)	4 (100.0)	0 (0.0)	0.569
	Absense (*N* = 57)	40 (70.2)	17 (29.8)	
Preoperative	≦37 U/ml (*N* = 23)	18 (78.3)	5 (21.7)	0.539
Serum CA19-9	>37 U/ml (*N* = 29)	20 (69.0)	9 (31.0)	
Preoperative	≦5 ng/ml (*N* = 38)	30 (78.9)	8 (21.1)	0.182
Serum CEA	>5 ng/ml (*N* = 15)	9 (60.0)	6 (40.0)	

*CA19-9* carbohydrate antigen 19-9, *CEA* carcinoembryonic antigen *MF* mass forming type, *PI* periductal infiltrating type.

^a^13 patients records were not available.

^b^Differences between continuous variables were determined using the Mann–Whitney U test. Categorical variables were compared by Fisher’s exact test.

### Suppressed immune milieu in ICC is associated with that of tumour-draining lymph nodes

**Fig. 4:**
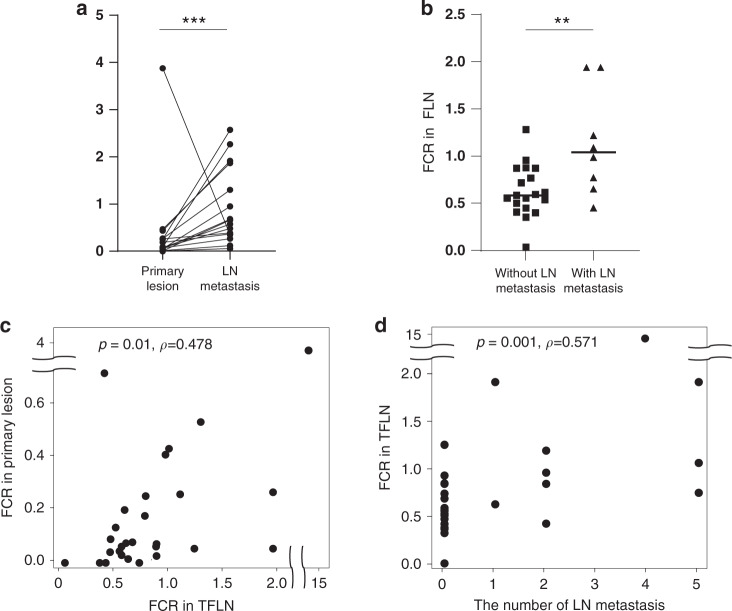
FoxP3^+^/CD8^+^ TIL ratio of primary lesions and lymph nodes. **a** Dot plot showing that the FoxP3^+^/CD8^+^ TIL ratio (FCR) is higher in metastatic lymph nodes than in primary lesions. ***P < 0.001. **b** Dot plot showing that the FCR in TFLN is higher in patients with ICC and lymph node metastasis than in those without lymph node metastasis. One FCR value in patients with ICC and lymph node metastasis was not shown in this figure because of outlier. TFLN tumour-free lymph node, **P < 0.01. **c** Dot plot showing the relatively strong correlation between the FCR in TFLN and that in the primary lesions (*P* = 0.01, rho = 0.478, Spearman’s rank correlation coefficient). **d** Dot plot showing the relatively strong correlation between the FCR in TFLN and the number of metastatic lymph nodes (*P* = 0.001, rho = 0.571, Spearman’s rank correlation coefficient).

The association of high FCR with pathological lymph node metastasis raised the possibility that the suppressed immune milieu in the primary lesion might promote lymph node metastasis. To clarify the biological role of FCR in lymph node metastasis in patients with ICC, we determined the FCR of lymph node specimens obtained from 47 patients with ICC who underwent lymphadenectomy in the same clinical validation cohort. First, we compared the FoxP3^+^ TILs, CD8^+^ TILs and FCR of metastatic lymph nodes with those of the corresponding primary lesions. Foxp3^+^ TILs were more and CD8^+^ TILs were less accumulated in metastatic lymph nodes than in the primary tumour (*P* < 0.01 and *P* < 0.01, respectively, Wilcoxon signed-rank test, Supplementary Fig. 4A and 4B). As expected, the FCR of metastatic lymph nodes was significantly higher than that of the primary lesions (*P* < 0.001, Wilcoxon signed-rank test; Fig. 4a), implicating a more advanced suppression of the immune milieu of metastatic lymph nodes compared with that of the primary lesion. Next, we determined whether the immune milieu of regional lymph nodes was suppressed prior to metastatic involvement by ICC by comparing the FCR of tumour-free lymph nodes (TFLN) of patients with and without lymph node metastasis. Intriguingly, the FCR of the TFLN in patients with lymph node metastasis was significantly higher than that of the TFLN in the patients without lymph node metastasis (*P* = 0.003, Mann–Whitney *U* test; Fig. 4b). Additionally, there was a strong correlation between the FCR of the TFLN and that of the primary lesion (*P* = 0.01, rho = 0.478, Spearman’s rank correlation coefficient; Fig. 4c). Moreover, there was a relatively strong correlation between the FCR of the TFLN and the number of metastatic lymph nodes (*P* = 0.001, rho = 0.571, Spearman’s correlation test; Fig. 4d). These results suggested that the suppressive immune milieu in ICC promoted lymph node metastasis by forming a premetastatic niche in TFLN.

## Discussion

In the present study, we utilised comprehensive approaches based on gene and protein expression analyses to demonstrate the clinical relevance of FCR in ICC. Our analyses revealed several key findings. First, we identified the characteristics of highly immunogenic ICCs based on immunophenoscore analysis. Second, among the effector and suppressor cells and immune checkpoint and MHC-related molecules, intratumoral CD8^+^ and FoxP3^+^ TIL counts were the best indicators of local response in highly immunogenic ICCs; these markers tended to have a prognostic impact. Third, a high FCR might be considered a predictor of poor OS in patients with ICC. Finally, a suppressed immune milieu in ICC might promote lymph node metastasis by facilitating the formation of a premetastatic niche.

We found that FoxP3^+^ TILs induced a suppressive local immune milieu in highly immunogenic ICCs. In our initial metagene analysis, CD8^+^ and FoxP3^+^ TILs, TIM-3 and HLA-A emerged as key components of highly immunogenic ICCs. TIM-3, which is expressed on the surface of CD4^+^ and CD8^+^ T cells, is involved in enforcing T-cell exhaustion by interaction with tumour cells [24]; therefore, TIM-3 is considered as an exhaustion marker of lymphocytes similar to programmed cell death 1 (PD-1). On the other hand, FoxP3 is primarily expressed in regulatory T cells (Treg), which induce a suppressive immune milieu [25]. Interestingly, single-cell transcriptomic analysis in ICCs has revealed that CD8^+^ T cells express TIM-3 and that FoxP3^+^ T cells display highly immunosuppressive characteristics [26], consistent with our metagene analysis. In contrast, in the current study the CD8^+^ TIL count, which did not correlate with TIM-3 expression, tended to associate with FoxP3^+^ TIL, suggesting that FoxP3^+^ TILs induce a suppressive local immune milieu in highly immunogenic ICCs. Recently, it is appreciated that Tregs not only suppress cells intratumorally via direct engagement but also serve as key interactors in the peritumour, stroma, vasculature and lymphatics to limit anti-tumour immune responses prior to tumour infiltration [27]. Scott et al. indicated that Tregs in intra-tumour inhibit the cytotoxic function of CD8^+^ TILs through dendritic cells or cytokines and that Tregs in tumour stroma blocks the infiltration of CD8^+^ TILs by inhibiting the formation of high endothelial venules (HEV). In this study, both TILs counts were higher in stroma than those in intra-tumour. These results suggest that ICC inhibits CD8^+^ TILs infiltrating to intra-tumour by inducing Tregs in the stroma.

In the current study, we also found that the ratio between FoxP3^+^ and CD8^+^ TILs, i.e. the FCR, was associated with poor prognosis and lymph node metastasis in ICC. To date, not only the CD8^+^ and FoxP3^+^ TIL counts but also the FCR has been reported as prognostic predictors in several malignancies, such as non-small cell lung carcinoma, breast cancer, and gastroenterological malignancies [28–30]. In addition, the utility of FCR as a predictor of response to chemotherapy has been shown in breast cancer [23, 31]. In these studies, a higher number of FoxP3^+^ TILs in the tumour immune milieu compared with the number of CD8^+^ TILs was shown to contribute to poor OS and refractoriness to chemotherapy [23, 31]. Additionally, Jing et al. demonstrated that the recurrence-free survival and OS were worse in ICCs with a higher FCR compared to those with a lower FCR [12] in agreement with our findings, supporting the hypothesis that FCR is a prognostic marker for poor OS in ICC.　In this study, patients with high FoxP3^+^ TIL count in intra-tumour of ICC have poor survival. These results suggest that inhibition of cytotoxicity of CD8^+^ TILs through FoxP3^+^ TILs in intra-tumour could more affect the tumour biology than blockade of CD8 + infiltrating to intra-tumour by Treg in the stroma.

To the best of our knowledge, this is the first study showing that FCR is associated with lymph node metastasis in ICC. While there have been no reports concerning about immunogenic meaning of FCR in the metastatic lesion and primary tumours, several studies focusing on CD8^+^ TILs in the metastatic lesion and the corresponding primary tumour in breast cancer have been reported [32, 33]. Ogiya et al. demonstrated that CD8^+^ TILs were less accumulated in metastatic sites than in the corresponding primary tumour, and concluded that immune escape could accelerate tumour progression [32]. In this study, CD8^+^ TILs were also less observed in metastatic lymph nodes than in the primary tumour (*P* < 0.01, Wilcoxon rank-sum test). This result was concordant with the previous reports, and indicated that the tumour cells escape from immune surveillance. In addition, Zhou et al. illustrated that tumour-infiltrating Tregs selectively migrate to tumour-draining lymph nodes [34]. Indeed, in the current study, FoxP3^+^ TILs were more accumulated in metastatic lymph nodes than in the primary tumour (*P* < 0.01, Wilcoxon rank-sum test). High FoxP3^+^ TIL counts were reported in metastatic lymph nodes in other malignancies such as non-small cell lung carcinoma, breast cancer and cervical cancer [35–38]. Intriguingly, Heeren et al. revealed that cervical cancers with lymph node metastases harboured a high FCR not only in tumour-positive lymph nodes but also in adjacent tumour-negative lymph nodes [37] and suggested that the delineated fields of regulatory T lymphocyte-associated immune suppression in anatomically co-localised lymph nodes receiving tumour drainage enabled metastasis by creating metastatic niches [37]. Similarly, our analyses revealed that the FCR was higher in the TFLN of patients with lymph node metastasis compared to those without lymph node metastasis, suggesting that the FCR was involved in niche formation in tumour regional lymph nodes close to the tumour. Lymph node metastasis is considered one of the strongest prognostic factors of ICC [39], and ICC with lymph node metastasis is considered a systemic disease according to the guidelines for the diagnosis and management of ICCs [40]. Our findings suggest that lymph node metastasis in ICC reflects a high metastatic potential mediated through immune suppression induced by FoxP3^+^ TILs.

Recent efforts to improve prognosis and OS rates in patients with malignancies have been devoted to the development of therapeutic approaches targeting FoxP3^+^ TILs with immune checkpoint blockade [41]. Although these approaches remain challenging, FoxP3^+^ TILs could be a target for therapy in the future.

In the present study, the small sample size due to the rarity of ICC was a major limitation. Additionally, this was a single-centre study. However, the analysis of the immune milieu in the primary tumour as well as in the corresponding lymph nodes was a strength of our study. We also acknowledge that the cut-off CD8^+^ and FoxP3^+^ TIL counts remain to be determined similarly to an issue with previous studies. Further studies in larger cohorts are needed to validate the utility of CD8^+^ and FoxP3^+^ TIL counts as prognostic and predictive biomarkers in ICC.

In conclusion, we used complementary analyses to demonstrate the potential utility of intratumoral FCR as a prognostic marker in ICC. Furthermore, this is the first study to reveal that the balance of CD8^+^ and FoxP3^+^ TILs might contribute to ICC progression by forming a premetastatic niche in lymph nodes. These data highlight the critical role the FCR might play in cancer biology and suggest that CD8^+^ and FoxP3^+^ TILs might be considered therapeutic targets in ICC.

## Supplementary information

Supplementary data

BJC-A3339392 checklist

## Data Availability

The datasets used and/or analysed during the current study are available from the corresponding author on reasonable request.
