# Supplementary material for: Regulatory T cells induce a suppressive immune milieu and promote lymph node metastasis in intrahepatic cholangiocarcinoma
Source: Br J Cancer. 2022 May 21;127(4):757–65. doi: 10.1038/s41416-022-01838-y (PMC9381563; doi:10.1038/s41416-022-01838-y)
Supplement: Supplementary file 1 — Supplementary data [file 41416_2022_1838_MOESM1_ESM.docx]

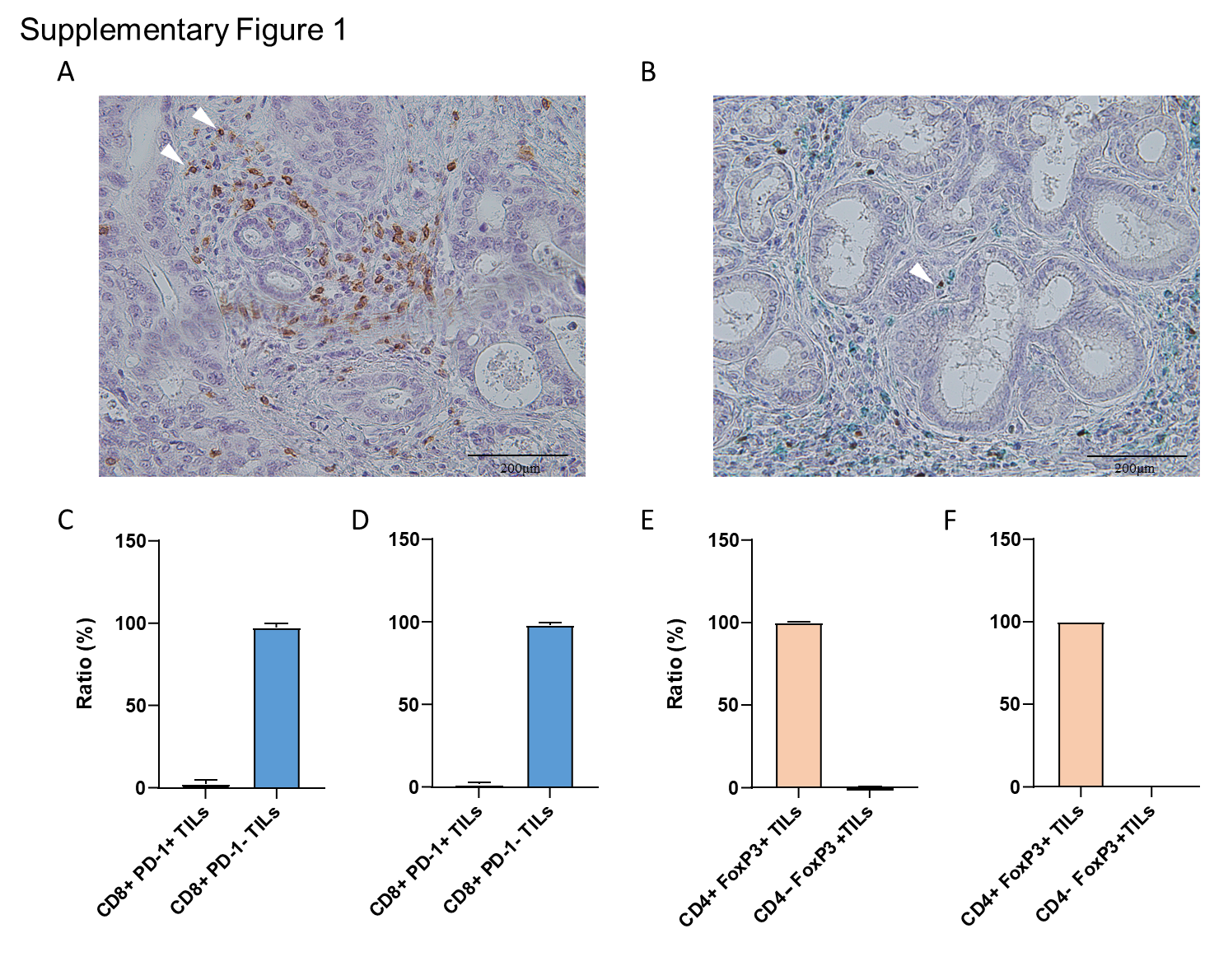


**Supplementary Figure 1**: Enzymatic double staining immunohistochemistry to analyze the phenotypes of TILs in ICCs. **(A)** White arrowhead shows CD8+ PD-1+ TILs in ICCs. CD8 and PD-1 on cell membrane of lymphocytes is stained with brawn and green, respectively. **(B)** White arrowhead shows CD4+ FoxP3+ TILs in ICCs. CD4 on cell membrane of lymphocytes and FoxP3 within nuclear is stained with green and brawn, respectively. Lower left bar graphs show the distribution of **(C)** intratumoral and **(D)** stromal CD8+ PD-1+ TIL counts of nine ICC patients. Lower right bar graphs show the distribution of **(E)** intratumoral and **(F)** stromal CD8+ PD-1+ TIL counts of nine ICC patients.


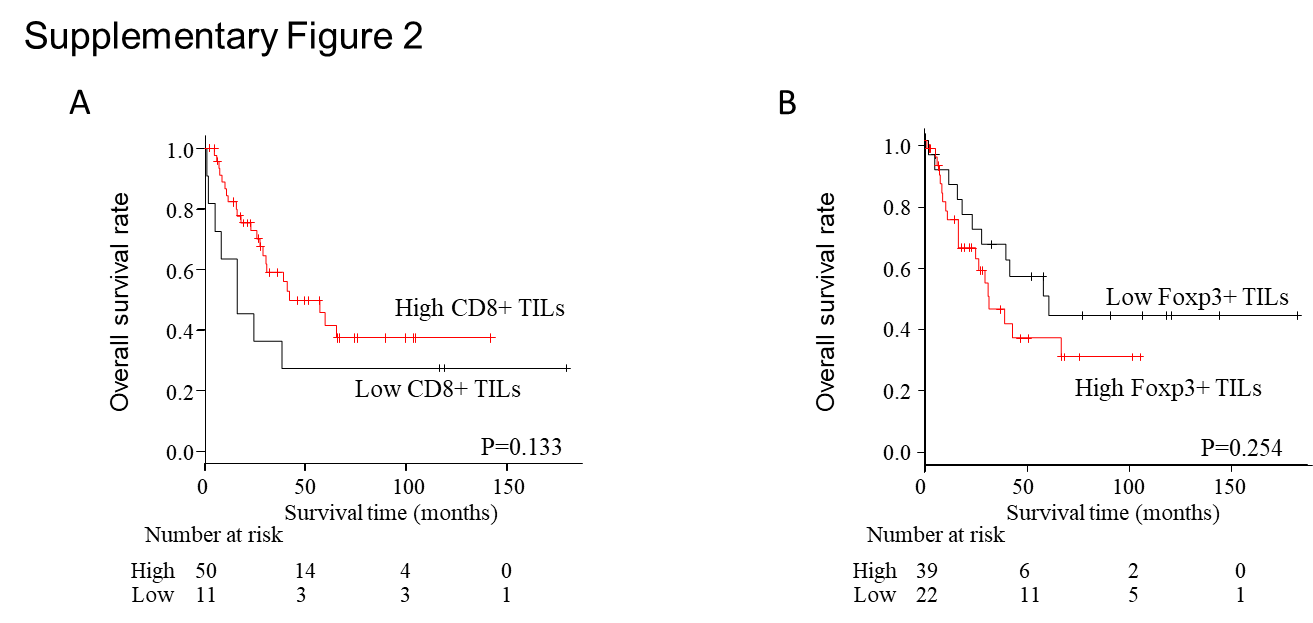


**Supplementary Figure 2**: Prognostic analysis with tumor infiltrating lymphocytes (TILs) in stroma of ICCs

Overall survival analysis were conducted based on (A) CD8+ TILs and (B) Foxp3+ TILs in stroma of ICCs.


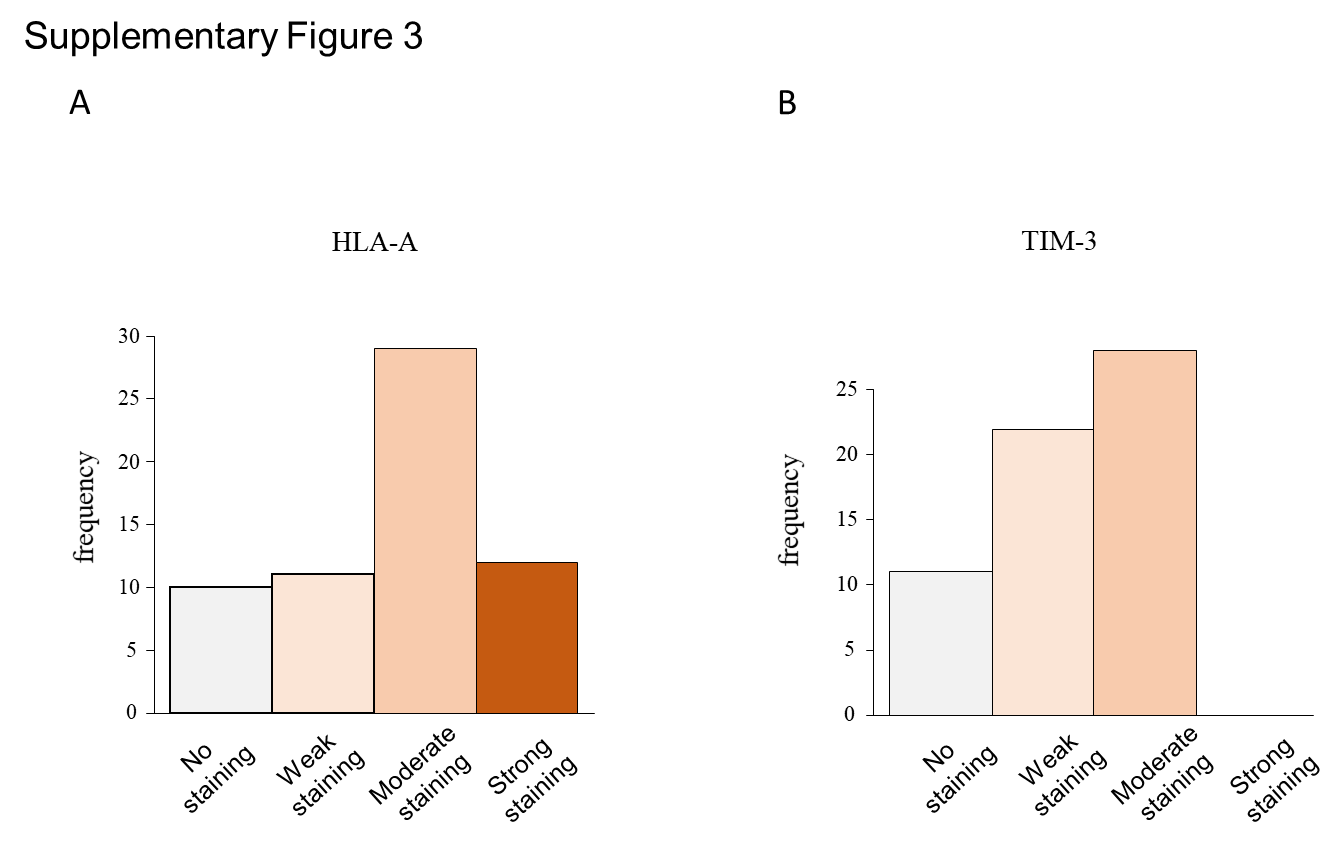


**Supplementary Figure 3**: Distribution of protein expression according to Immunoreactive score (IRS)

(A) HLA-A and (B) TIM-3 protein expression was evaluated in the tumor cell and lymphocytes, respectively. Protein expression was categorized into four subsets by IRS score as follows: 0–1 = no staining, 2–3 = weak staining, 4–8 = moderate staining, and 9–12 = strong staining.


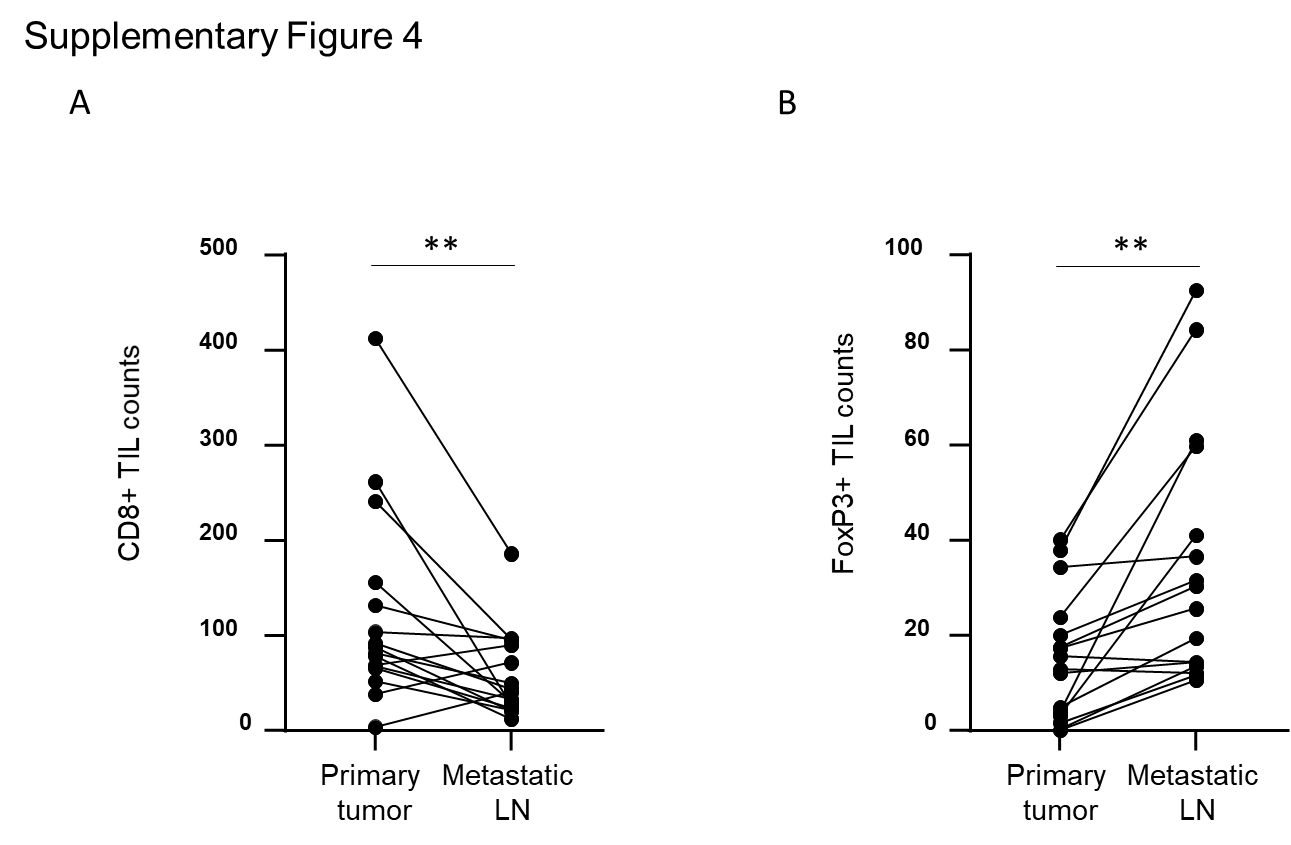


**Supplementary Figure 4**: CD8^+^ and FoxP3 TILs in primary lesions and metastatic lymph nodes. **(A)** Dot plot shows that the CD8^+^ TILs are less observed in metastatic lymph node than in the corresponding primary. LN; lymph node, ** *P* < 0.01. **(B)** Dot plot shows that the FoxP3+ TILs are more accumulated in metastatic lymph node than in corresponding primary lesion. LN; lymph node, ** *P* < 0.01.

| **Supplementary Table 1 The information of primary antibody for IHC analysis** |
| --- |
| mouse anti-CD8 monoclonal antibody (GTX72053, GeneTex, CA, USA; dilution 1;200) |
| Anti-PD1 antibody [CAL20] (ab237728, abcam, Cambridge, UK; dilution 1:200) |
| mouse anti-FOXP3 monoclonal antibody (ab20034, abcam, Cambridge, UK; dilution 1:100), |
| Anti-CD4 antibody [RM1013] (ab288724, abcam, Cambridge, UK; dilution 1:500) |
| human TIM3-antibody (AF2365, R&D systems, MN, USA; dilution 1:500) |
| Anti-HLA A Antibody (ab52922, abcam, Cambridge, UK; dilution 1:500) |

| **Supplementary Table 2: Correlation between CD8+ TILs and other three variables** | | | | | |
| --- | --- | --- | --- | --- | --- |
| Variables | | Total | Low CD8+ TIls | High CD8+ TILs | P value |
|  |  | N=61 | (N=44) | (N=17) |  |
| **FoxP3+ TILs** |  |  |  |  |  |
|  | Low | 40 | 16 (40.0%) | 24 (60.0%) | 0.046 |
|  | High | 21 | 3 (14.3%) | 18 (85.7%) |  |
| **HLA-A** |  |  |  |  |  |
|  | No | 9 | 5 (55.6%) | 4 (44.4%) | 0.075 |
|  | Weak | 11 | 2 (18.2%) | 9 (81.8%) |  |
|  | Moderate | 29 | 11 (37.9%) | 18 (62.1%) |  |
|  | Strong | 12 | 1 (8.3%) | 11 (91.7%) |  |
| **TIM-3** |  |  |  |  |  |
|  | No | 11 | 5 (45.5%) | 6 (54.5%) | 0.356 |
|  | Weak | 22 | 5 (22.7%) | 17 (77.3%) |  |
|  | Moderate | 28 | 9 (32.1%) | 19 (67.9%) |  |
|  | Strong | 0 | 0 (0.0%) | 0 (0.0%) |  |
| Categorical variables were compared by Fisher’s exact test. | | | | | |
